# Antifungal potential of volatiles produced by *Bacillus subtilis* BS-01 against *Alternaria solani* in *Solanum lycopersicum*

**DOI:** 10.3389/fpls.2022.1089562

**Published:** 2023-01-26

**Authors:** Zoia Arshad Awan, Amna Shoaib, Peer M. Schenk, Ajaz Ahmad, Saleh Alansi, Bilal Ahamad Paray

**Affiliations:** ^1^ Faculty of Agricultural Sciences, University of the Punjab, Lahore, Pakistan; ^2^ Plant-Microbe Interactions Laboratory, School of Agriculture and Food Sciences, University of Queensland, Brisbane, QLD, Australia; ^3^ Department of Clinical Pharmacy, College of Pharmacy, King Saud University, Riyadh, Saudi Arabia; ^4^ Botany and Microbiology Department, College of Science, King Saud University, Riyadh, Saudi Arabia; ^5^ Zoology Department, College of Sciences, King Saud University, Riyadh, Saudi Arabia

**Keywords:** biological agent, GC-MS, secondary metabolites, pathogen load, qPCR

## Abstract

Bacterial biocontrol agent/s (BCAs) against plant diseases are eco-friendly and sustainable options for profitable agricultural crop production. Specific beneficial strains of *Bacillus subtilis* are effective in controlling many fungal diseases including Alternaria blight caused by a notorious pathogen “*Alternaria solani*”. In the present study, the biocontrol attributes of a newfangled strain of *B. subtilis* (BS-01) have been investigated and its bioactive compounds were also identified against *A. solani*. The volatile organic compounds (VOCs) produced by BS-01 in organic solvents viz., *n*-hexane, dichloromethane, and ethyl acetate were extracted and their antifungal efficacy has evaluated against *A. solani.* Also, the preventive and curative biocontrol method to reduce the fungal load of *A. solani* was estimated by both foliar and seed applications on infected tomato (Solanum lycopersicum) plants as determined by quantitative PCR assays. Growth chamber bioassay revealed that both foliar and seed application of BS-01 on tomato plants previously or subsequently infected by *A. solani* significantly reduced the pathogen load on inoculated tomato foliage. Results showed that antifungal bioassays with various concentrations (10-100 mg mL^-1^) of extracted metabolites produced by BS-01 in ethyl acetate fraction showed the highest inhibition in fungal biomass (extracellular metabolites: 69-98% and intracellular metabolites: 48-85%) followed by *n*-hexane (extracellular metabolites: 63-88% and intracellular metabolites: 35-62%) and dichloromethane (extracellular metabolites: 41-74% and intracellular metabolites: 42-70%), respectively. The extracted volatile compounds of BS-01 were identified *via* GC-MS analysis and were found in great proportions in the organic fractions as major potent antifungal constituents including triphenylphosphine oxide; pyrrolo[1,2-a] pyrazine-1,4-dione, hexahydro-3-(2-methylpropyl); pyrrolo[1,2-a] pyrazine-1,4-dione, hexahydro-3-(phenylmethyl); *n*-hexadecanoic acid; *n*-tridecan-1-ol; octadecane; octadecanoic acid; eicosane and dodecyl acrylate. Separate or mixture of these bioactive VOCs had the potential to mitigate the tomato early blight disease severity in the field that would act as a sustainable plant protection strategy to generate profitable tomato production.

## 1 Introduction

Biological control of plant diseases using bacterial-based biocontrol agents is considered a safer and more sustainable alternative over synthetic pesticides (Daranas et al., 2019). Many microbial biopesticides can also act as biofertilizers that contribute to nutrient cycling, enhance soil fertility, and improve crop yields (Olanrewaju et al., 2017). Multiple mechanisms have been identified for BCAs, including hyperparasitism, competition with plant pathogens (e.g. *via* the production of siderophores), priming leading to induced systemic resistance, and direct antimicrobial actions such as inactivation of pathogen enzymes, production of antibiotics, lytic enzymes (cellulase, chitinase, and proteases) or toxins (Oliva et al., 2017; Khanna et al., 2019a). For example, numerous species of *Bacillus*, *Streptomyces* and *Pseudomonas* have been identified as plant-growth-promoting bacteria (PGPRs) and biocontrol agents (BCAs) against early blight (EB) (Moreira et al., 2014; Manimaran et al., 2017; Wang et al., 2018; Khanna et al., 2019b).

The genus *Bacillus* has a unique ability to replicate promptly and exhibit broad-spectrum antibiotic activity (Shafi et al., 2017; Syed-Ab-Rahman et al., 2018; Shoaib et al., 2019). Biopesticides formulated from various strains of *Bacillus* such as *B. subtilis*, *B. sphaericus* and *B. thuringiensis* have a positive effect on plant growth by inducing systematic acquired resistance (SAR) in the host and inhibiting disease-causing pathogens (Lastochkina et al., 2019; Rashid et al., 2022). Such novel beneficial biological agents including *Bacillus* and *Paenibacillus* species are reported to improve the nutritional values of staple crops and could be used as bio-inoculants (Hussain et al., 2020; Ilyas et al., 2022). Amongst various species of *Bacillus*, *Bacillus subtilis* is widely distributed and one of the most attractive bio-agent, which is easy to isolate and culture (Zhang et al., 2016). *B. subtilis* is widely used to control agricultural diseases due to its good antimicrobial activity in the soil and strong adaptability and (Chandrasekaran et al., 2016). *B. subtilis* has been known to produce antimycotic enzymes viz., chitinase, cellulose and beta-1,3 glucanase by degrading fungal structural polymers (Yánez-Mendizábal et al., 2011). It is also considered one of the most widely used and well-studied biocontrol organisms, and 4-5% of its genome is responsible for the synthesis of antibiotics including lipopeptides, Iturin, surfactin and fengycin that contribute to the antifungal potential, for example, lipopeptides have shown low environmental toxicity and high biodegradability characteristics (Guo et al., 2014). Hence, such antibiotics are eco-friendly and environmentally sustainable as compared to chemical pesticides (More et al., 2014). Therefore, *B. subtilis* has been affirmed safe by the US Food and Drug Administration in the food processing industries (Mnif and Ghribi, 2015). Nowadays, numerous *B. subtilis*-based commercial products such as AvoGreen, Bio Yield, BioSafe and Ecoshot, etc., are available to manage many fungal diseases (Lastochkina et al., 2019; Hashem et al., 2019). Besides, *B. subtilis* strains also accelerate phosphate solubilization, nitrogen uptake, siderophore and phytohormone for better plant growth and development (Gouda et al., 2018);. Likewise, its antifungal potential against a broad range of phytopathogens has been confirmed *in vitro* and *in vivo* (greenhouse and field) studies (Hadimani and Kulkarni, 2016; Shoaib et al., 2019; Awan and Shoaib, 2019). The tolerance and resilience against plant disease are primarily related to genetics, several of the traits are required for a number of the mechanisms for biocontrol in for the synthesis of polyamines, the production of siderophores, and the synthesis of antimicrobial peptides and antibiotics which are directly involved in plant defense as structural components (e.g. thickness of cell walls) and metabolic regulators (e.g. antioxidants, phytoalexins and flavonoids Where, practical implementation of *B. subtilis* strain/s to manage tomato early blight may wean off dependence on agricultural chemicals against notorious pathogen *A. solani* (Köhl et al., 2019).

The present study aimed to isolate, identify and characterize a new strain of *Bacillus subtilis* (BS-01). Extracted volatiles from extra- and intra-cellular metabolites of BS-01 will be examined for antifungal impact and identified using GC-MS analysis. The capability of reducing Alternaria pathogen load on pathogen-inoculated tomato foliage also will be estimated by employing BS-01 (*in vivo* trial). Employing BS-01 as an alternative approach is likely to lead to a more rational and sustainable choice of disease management for promoting plant growth.

## 2 Material & methods

### 2.1 Isolation and cultivation of microbial strains

- ***Isolation of pathogen:*** *A. solani* a pathogen of tomato early blight pathogen (FCBP 1401; MF539619) was re-isolated from infected tomato leaves showing the characteristic disease symptoms of EB following the protocol of Shoaib et al., 2019. For *in vitro* and *in vivo* bioassays the conidial suspension was prepared and used for further study (Awan et al., 2018).
- ***Isolation of biocontrol agent:*** A beneficial strain of *Bacillus subtilis* was isolated from the rhizospheric soil of a chickpea field in the experimental area of the mother institute University of the Punjab, Lahore, Pakistan. For the microbial cultivation, about 1 g of soil samples were serially diluted 10-folds in phosphate-buffered saline (PBS, 0.05 M, pH 7.4) and 100 μL of soil suspensions were plated on Luria-Bertani agar (LB) (1% tryptone, 1% NaCl 1%, 0.5% yeast extract 0.5% with pH 7.5). After incubation of 24 h at 37°C, single colonies were picked and maintained as pure cultures on LBA plates and the pure culture of *B. subtilis* (Genebank accession LC425129.1) was maintained in LB with 20% glycerol for long-term storage at -80°C.

### 2.2 Evaluation of *B. subtilis* (BS-01) to reduce the pathogen load

A pot assay was conducted in a growth chamber to assess the biocontrol efficacy of *B. subtilis* in reducing fungal load by preventive (pre-infection) and curative (post-infection) methods (Syed-Ab-Rahman et al., 2019). The experiment has been repeated thrice, comprised of six treatments (T1-T6) and arranged in a completely randomized design with five replications (N=5) of each treatment. The treatments (T_1_-T_6_) of pot bioassays were described in **Table 1**. Where, T_1_: -ve control (healthy tomato plants without any treatment and inoculation), T_2_: +ve control (plants inoculated with AS only), T_3_ & T_4_: pre-infection treatments and T_5_ & T_6_ were post-infection treatments.

**Table 1:** *In vivo* experimental design to check the control efficacy of *B. subtilis* (BS-01) against *Alternaria solani*.

Treatments	Description
**T1**	Healthy tomato plants without any treatment and inoculation, sprayed with distilled water only (-ve control)
**T2**	Tomato plants inoculated with the *A. solani* only (+ve control)
**T3**	Tomato seeds treated with BS-01 (*B. subtilis*) before sowing (pre-infection treatment)
**T4**	Tomato foliage treated with BS-01 (*B. subtilis*), followed by inoculation with *A. solani* after 24 hours (pre-infection treatment)
**T5**	Tomato foliage was first inoculated with *A. solani*, followed by being treated with BS-01 (*B. subtilis*) after 24 hours (post-infection treatment)
**T6**	Tomato foliage was first inoculated with *A. solani*, and after 24 hours, rhizospheric soil supplemented with BS-01 (*B. subtilis*) (post-infection treatment)

A suspension of BS-01 was prepared by harvesting bacterial cells from a one-day-old bacterial culture in ice-cold 0.1M PBS (pH 6.8). Tomato seeds were surface-sterilized with 1% bleach for 2 min followed by washing in 70% (v/v) ethanol for 5 min and rinsing three times with distilled water. Before seed sowing, dried sterilized tomato seeds were soaked in 0.05 M phosphate-buffered saline (PBS; pH: 6.8) for all treatments, but tomato seeds for treatment T_3_ were soaked in BS-01 suspension (OD595_nm_ = 0.8) [prepared by harvesting bacterial cells (BS-01) from a 24-hours old culture in ice-cold 0.05 M phosphate-buffered saline (PBS, pH: 6.8)] for 30 min as a pre-infection treatment (preventive measure against EB). Tomato seeds (1 seed pot^-1^) were sown in pots (3.15"height × 3.15" width) and incubated in a growth chamber (16 hours daylight at 28°C and 8 hours a night at 20°C). After twenty days, tomato plants of all treatments were inoculated with 1-2 mL of conidial suspension (2.0 × 10^4^ conidia mL^-1^) through a hand sprayer. But, plants in treatment T_4_ were treated with 1-2 mL of BS-01 suspension (OD595_nm_ = 0.8) a day before pathogen inoculation (*A. solani*) as a pre-infection treatment, while plants in treatment T_5_ were treated with 1-2 mL of BS-01 suspension (OD595_nm_ = 0.8) a day after pathogen inoculation (*A. solani*) as a post-infection treatment. In treatment T_6_, the root surrounding soil (rhizosphere soil) of plants was supplemented with BS-01 suspension (OD595_nm_ = 0.8) a day after pathogen inoculation. All the pots were placed in trays which were filled with distilled water to maintain soil moisture (40-50%). The plants were harvested after 15 days of pathogen/bacterial inoculation (30-days old plant) and the reduction in fungal load of *A. solani* due to different inoculation methods was quantified by real-time quantitative PCR (qPCR) (Awan et al., 2022).

#### 2.2.1 DNA extraction of tomato leaves

DNA of the tomato leaves from the above treatments was isolated 2 weeks after pathogen/bacterial inoculation. A modified CTAB (cetyltrimethyl ammonium bromide) protocol was used for DNA extraction from 0.25 g of leaf sample ground in liquid nitrogen and mixed with 600 µL of 2% CTAB buffer (2 g CTAB, 10 mL of 1 M Tris-HCl, 4 mL of 0.5 M EDTA, 28 mL of 5 M NaCl, 2 mL of beta-mercaptoethanol and 56 mL of autoclaved distilled water) in a pre-cooled Eppendorf tube. After incubation (60°C 1 h) and centrifugation (13,000 rpm for 10 min), the resulting supernatant was mixed with chloroform and isoamyl alcohol (24:1). The topmost aqueous phase was separated and successively mixed with 50 µL of 3 M sodium acetate and 500 µL of absolute ethanol (100%), incubated (-20°C 1 h) and centrifuged (13,000 rpm for 20 min) for the precipitation of DNA. The DNA pellet was washed thrice with 300 µL ethanol (70% v/v) and the dried pellet was re-suspended in TE buffer (Tris-EDTA buffer [0.2 mL of 0.5 M EDTA and 1 mL of 1 M Tris-HCl pH 8.0] to preserve it at -20°C (Syed-Ab-Rahman et al., 2018). The extracted genomic DNA was quantified and adjusted to 20 ng µL^-1^ for qPCR amplification using a Thermo Scientific NanoDrop apparatus.

#### 2.2.2 Real-time quantitative PCR

For pathogen quantification, a set of *Alternaria solani*-specific primers for cytochrome *b* As_Cytb_F (5′-TCA GGA ACT CTG TGG CGT ATC-3′) and As_Cytb_R (5′-TCA GAT GAA AGG GAG GGA GGA C-3′) and another set of primers for a tomato house-keeping gene *ACTIN* Act_F (5′-GGC AGG ATT TGC TGG TGA TGA TGC T-3′) and Act_R (5′-ATA CGC ATC CTT CTG TCC CAT TCC GA-3′) were used.

For qPCR, 10 µL of the reaction mixture contained 5 µL SYBR green, 0.7 µL of each primer (10 µM), and 3.6 µL of extracted DNA (20 ng mL^-1^). Quantitative PCR (qPCR) was performed using a CFX96 Touch™ Real-Time PCR detection system (Life Science Research, Bio-Rad). Thermal cycling conditions were set as follows: initial denaturation for 2 min at 95°C, followed by 5 s at 95°C and 10 s at 60°C for 45 cycles, and a final extension step at 60°C and 95°C for 5 s. Results were analyzed by the inbuilt software (CFX Manager™ software) connected to the CFX96 Touch™ Real-Time PCR detection system.

### 2.3 Identification and characterization of BS-01

Based on the previously reported studies (Shoaib et al., 2019; Awan and Shoaib, 2019 and Awan et al., 2022), this strain (BS-01) has antifungal potential against early blight pathogen. Thus, further identification and characterization of BS-01 were done in this study. *B. subtilis* (BS-01) was identified through standard protocols of phenotypic, biochemical and 16S rDNA gene sequencing. Phenotypic characterization was done by assessing the morphology of the colony and cell growth. Biochemical characterization was assessed by employing standard protocols and biochemical kit (Microgen biochemical identification kit) for Gram staining, oxidase activity, catalase activity, nitrate reduction, hydrolysis of gelatin, utilization of citrate, catalysis of malonate, production of acid from sugars (i.e., glucose, sucrose, lactose, arabinose, rhamnose, or raffinose) and production of alcohol sugars.

For 16S rDNA gene amplification, the chromosomal DNA was isolated using a bacterial DNA extraction kit (Genomic DNA mini kit, Thermo Fisher Scientific, USA) following the manufacturer’s instructions. Amplification by PCR was performed using universal primers 27f (5’AGAGTTTGATCCTGGCTCAG-3’) and 1492r (5’-TACGGTTACCTTGTTACGACT-3’) (Hadi, 2013). PCR amplification was carried out with a program as follows: initial denaturation at 94°C for 6 min, followed by 60 s at 94°C, 60 s at 56°C and 60 s at 72°C for 30 cycles and final extension for 10 min at 72°C. The amplified PCR product was purified using a GeneJET Gel Extraction Kit (Thermo Fisher Scientific) and sent for sequencing to Macrogen (South Korea). The obtained sequences were searched for homology with sequenced genes from the National Center for Biotechnology Information (NCBI) database. The DNAMAN bioinformatics tool was used to construct a phylogenetic tree sequence alignment of bacterial DNA.

### 2.4 Extraction and fractionation of bacterial metabolites

*B. subtilis* (BS-01) is used to extract the VOCs in the extracellular metabolites and intracellular cellular metabolites, separately in three different organic solvents of varying polarity viz., *n*-hexane, dichloromethane and ethyl acetate at the Plant-Microbe Interactions Laboratory, University of Queensland, Australia.

#### 2.4.1 Extracellular metabolites

The primary culture of *B. subtilis* was prepared in 20 mL of LB broth and kept in a shaking incubator at 30°C, 150 rpm for 24 h. The resulting starter culture was inoculated into 500 mL of LB broth supplemented with 5 g tryptone, 5 g NaCl and 2.5 g yeast extract in 500 mL of distilled water using 2000 mL Erlenmeyer flasks incubated at 30°C with 120 rpm shaking. After 72 h of incubation, the cell-free culture (supernatant) was obtained by centrifugation at 14,000 rpm for 20 min. This cell-free culture medium was allowed to concentrate (four times reduced) in the oven at 40°C for 48 h. The resulting metabolite concentrate was sequentially extracted with double volume (500 mL) of *n*-hexane, dichloromethane and ethyl acetate, respectively. Primarily, concentrated culture (250 mL) and expected organic solvent (500 mL) was thoroughly homogenized by shaking at 150 rpm for 30 min and allowed to stand for 6-8 hours in a separating funnel (1000 mL). After getting a clear separation, the expected organic layer was separated very carefully and dried in a 1000 mL round bottle flask on a rotary evaporator at 40°C to finally collect a slimy mass of crude metabolites by following the protocol of Syed-Ab-Rahman et al. (2018).

#### 2.4.2 Intracellular metabolites

For the extraction of intracellular metabolites, the secondary culture of *B. subtilis* was prepared from a primary culture as mentioned above. The bacterial cells were harvested at the exponential growth phase (OD595_nm_ = 0.5) after 24 h (30°C) followed by centrifugation (14,000 rpm at 4°C) for 20 min (Meyer et al., 2010). The cell pellet was separated by discarding the cell-free culture and then washed with autoclaved distilled water to remove the excess culture medium. The bacterial pellet was weighed (2 g) and suspended in 30 mL of ice-cold 0.1 M PBS (pH 6.8). BS-01 cells in PBS were lysed for 50 min at 4°C through a cell disruptor (SONICS Vibra-Cell™ Ultrasonic Liquid Processors), which was programmed with successive disruption for 10 s followed by a pause for 10 s with 40% amplitude. This cell lysate (30 mL) expected organic solvent (500 mL) i.e., *n*-hexane, dichloromethane, and ethyl acetate were used to extract intracellular metabolites as described above (Syed-Ab-Rahman et al., 2018).

Extraction and fractionation of VOCs from BS-01 extra- and intracellular metabolites were sequentially done according to the polarity of three organic solvents, first with *n*-hexane followed by dichloromethane then followed by ethyl acetate.

### 2.5 Preparation of stock and testing concentrations

The concentrated extracts of both extra- and intracellular metabolites in three different organic solvents were used to prepare a stock concentration solution. A test stock concentration of 100 mg mL^-1^ was prepared by dissolving 1 mg of each extracted metabolite (slimy mass) in 1 mL of the respective organic solvent (*n*-hexane, dichloromethane and ethyl acetate) in separate glass sample vials (1.5 mL). Different concentrations of each extracted fraction were prepared from the stock solution (100 mg mL^-1^) by diluting serially with respective organic solvents to make final concentrations (10, 20, 40, 60, 80 and 100 mg mL^-1^).

#### 2.5.1 Antifungal bioassays with organic fractions

The organic fractions (*n*-hexane, dichloromethane, and ethyl acetate) of VOCs extracted from extra- and intracellular bacterial metabolites were assessed for their antifungal activity against *A. solani*. The antifungal activity of the fractions was tested using broth micro-dilution techniques in 96-well microtitre plates. For the bioassay, a microplate (96-well) was filled with 200 µL of malt extract broth (2% ME). Each well was supplemented with 10 µL of different concentrations (10, 20, 40, 60, 80 and 100 mg mL^-1^) of the desired fraction and the same well was inoculated with 10 µL of a conidial suspension (1.0 × 10^3^ conidia mL^-1^). Microplate wells were filled with 2% ME broth (200 µL), supplemented with 10 µL of relevant pure organic solvent (*n*-hexane, dichloromethane and ethyl acetate) and 10 µL of a conidial suspension, served as a negative control treatment (0 mg mL-1: without extracted organic fraction).

For the positive control treatment, wells were filled with 2% ME broth and inoculated with 10 µL of conidial suspension of *A. solani*, only. *In vitro* antifungal bioassay was tested thrice) arranged in a completely randomized design with five replications (N=5) for each treatment. After incubation for 48 h at 28°C, the harvested fungal biomass was dried and weighed. Percentage inhibition in fungal biomass was calculated over the positive control using the following formula (Shoaib et al., 2019).


$$\text{Percent inhibition }\left(\%\right)\;=\frac{\text{Control}-\text{Treatment}}{\text{Control}}\times \;100$$

#### 2.5.2 Gas chromatography−mass spectrometry (GC-MS) analysis

The organic fractions of both extra- and intracellular metabolites were carried out in a GC-MS system (Shimadzu Corporation, Kyoto, Japan) for analysis. The flow rate of carrier gas (helium) was set at 17.5 mL min^-1^ at a constant linear velocity of 42.7 cm s^-1^ with a split ratio of 1:10. The injector temperature and initial oven temperature were kept at 320°C and 100°C, respectively. The temperature gradient of 100-340°C (10°C min^-1^) and isothermal at 100°C (for 1 min) were programmed in the oven. The mass spectrometer was operated with an ion source temperature of 250°C and an interface temperature of 340°C. The analysis was performed in a full-scan mode with a mass range of 42-500 m/z and the run time was completed in 30 min (Syed-Ab-Rahman et al., 2018). For data processing, GC-MS Postrun analysis software was employed. The constituents of peaks were finally recognized after comparing them with available data in the NIST-14 mass spectrum library (National Institute of Standards and Technology, USA).

### 2.6 Statistical analyses

Significant differences (p ≤ 0.05) in plant disease resistance assays were determined by Student’s t-test. The relative performance of the data recorded from *in vitro* bioassays was compared after getting significant results in the analysis of variance (ANOVA) and their means were compared using Fisher’s protected least significant difference test (LSD) at p ≤ 0.05 using Statistix 8.1.

## 3 Results

### 3.1 Evaluation of *B. subtilis* to control early blight in tomato

**Figure 1:**
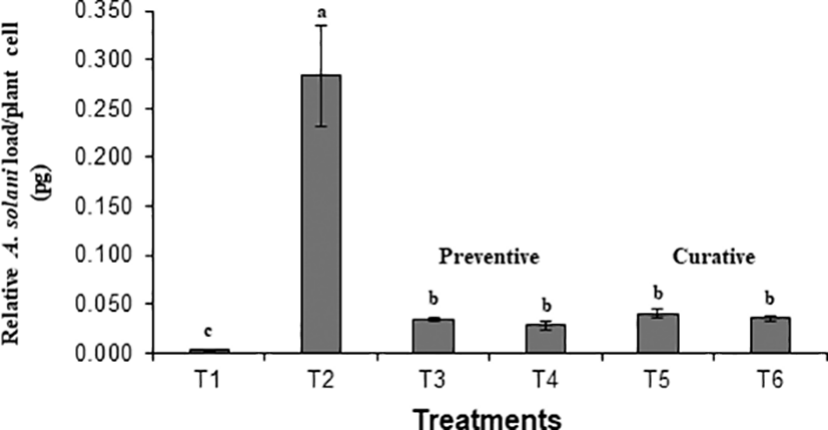
Effect of *Bacillus subtilis* (BS-01) on the relative fungal load of *Alternaria solani* (AS) in tomato foliage using qPCR after 7 days of pathogen inoculation. Values with different letters show a significant difference (p ≤ 0.05) mean value of five biological replicates (N=5) as determined by the LSD test. Error bars indicate the standard errors of the mean of replicates (N=5). Note. T_1_: -ve control (healthy tomato plants without any treatment and inoculation of AS), T_2_: +ve control (plants inoculated with AS only), T_3_: Tomato seeds treated with BS-01 (*B. subtilis*) before sowing, T_4_: Tomato foliage treated with BS-01 one day before AS inoculation; T_5_: Tomato foliage treated with BS-01 one day after AS inoculation; T_6_: tomato rhizosphere soil supplemented with BS-01 one day after AS inoculation.

Preventive and curative effects of BS-01 application to control tomato early blight were evaluated by quantifying pathogen load in tomato foliage using a set of *A. solani*-specific primers “cytochrome *b*”. The fungal load (0.283 pg) was highest in the positive control. It was assessed that both preventive and curative measures for the earl blight disease control in tomatoes significantly reduce the pathogen load by 85-90% as compared to the positive control (**Figure 1**). Notably, preventative measure (foliar or seed application with BS-01 before pathogen inoculation exhibited slightly more effective in reducing pathogen load as compared to curative measure (effect foliar or soil application with BS-01 following pathogen inoculation). Therefore, a significantly lowest fungal load by 90% (0.028 pg) was observed in a preventive method when plants were provided with a foliar application of BS-01 treatment before pathogen inoculation (T4) as compared to positive control plants.

### 3.2 Characterization of *Bacillus subtilis* BS-01

BS-01 was identified as *Bacillus subtilis* based on the morphological, biochemical and molecular data. It is a gram-positive, facultatively anaerobic and endospore-forming bacterium. Colonies on Luria-Bertani agar medium were medium-sized, white to creamy, dry, flat, and round with smooth margins. The bacterial cells were motile, rod-shaped, and occurred as small clusters and short chains. They displayed a positive reaction to catalase, oxidase, citrate, gelatin hydrolysis, malonate catalysis and the ability to ferment simple sugars (glucose and sucrose) as well as sugar alcohols (inositol, sorbitol, and adonitol) for acid production, but were negative for arabinose, rhamnose and raffinose (**Table 2**).

**Table 2:** Morphological and biochemical reactions of *Bacillus subtilis* (BS-01).

Cultural characters		Cell morphology		
Colony	White to creamy white, dry, flat, round with smooth margins	Shape		Rod
		Motile		+
Optimum temperature	30-37 °C	Gram type		+
		Endospore		+
Reactions		Reactions		
Catalase	+	Sorbitol		–
Oxidase	+	Adnonitol		–
Citrate	+	Arabinose		+
Gelatin hydrolysis	+	Raffinose		+
Malonate	+	Rhamnose		+
Inositol	–	Sucrose		+
		Lactose		–

**Figure 2:**
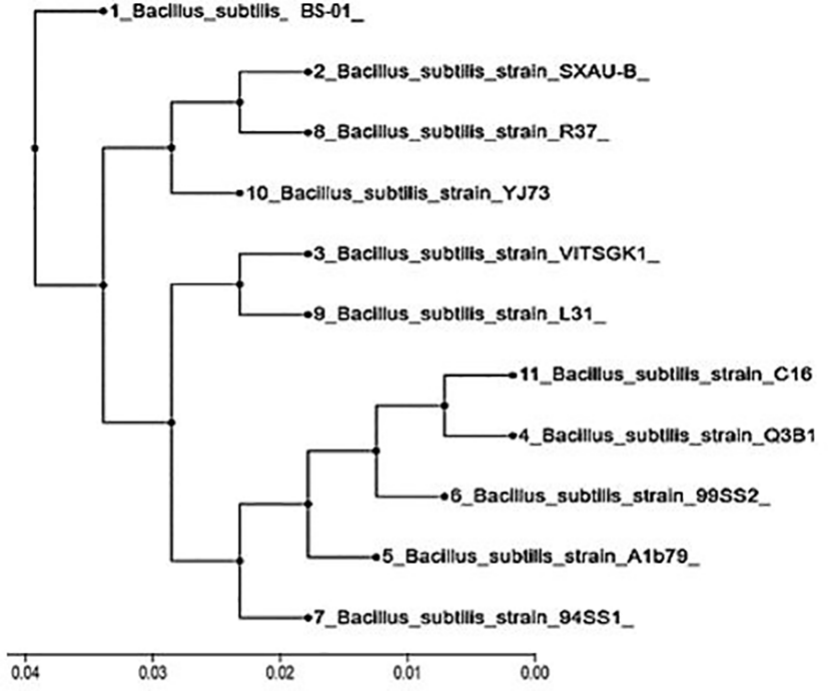
Phylogenetic tree constructed based on the 16S rDNA gene for *Bacillus subtilis* (BS-01) and ten other strains obtained from the NCBI database.

16S rDNA sequences of BS-01 were aligned and identified after blasting the sequence reads against the National Center for Biotechnology Information (NCBI) nucleotide database. A sequence of BS-01 was deposited in the NCBI GenBank database with the accession number LC425129.1. The alignment results showed that the strain BS-01 was closely related to the following *Bacillus subtilis* strains i.e., SXAU-B (MK875169.1), R37 (MK696406.1), YJ73 (KY652934.1), VITSGK1 (MK817557.1), L31 (KY652944.1), C16 (MH141058.1), Q3B1 (MK774698.1), 99SS2 (MK713722.1), A1b79 (MK737184.1) and 94SS1 (MK713700.1) with the highest similar identity 99.9–100% query cover. Finally, a phylogenetic tree was constructed using Clustal X analysis of MEGA7 (**Figure 2**).

### 3.3 Antifungal activity of extracellular and intracellular metabolites

**Figure 3:**
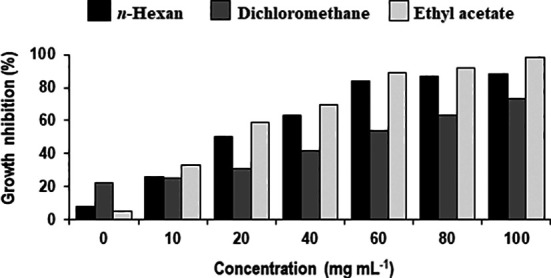
Percentage decrease in biomass of *Alternaria solani* due to different fractional concentrations of extracellular metabolites of *Bacillus subtilis* (BS-01).

**Figure 4:**
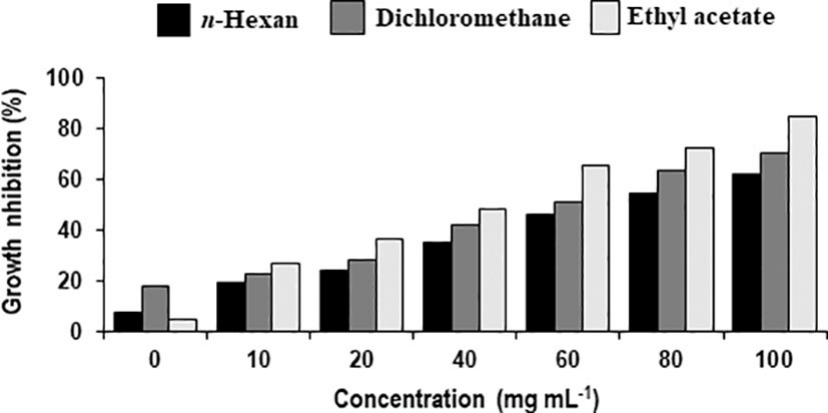
Percentage decrease in biomass of *Alternaria solani* due to different fractional concentrations of intracellular metabolites of *Bacillus subtilis* (BS-01).

Different concentrations of ethyl acetate, *n-*hexane and dichloromethane and fractions of extracellular metabolites significantly (p ≤ 0.05) decreased fungal biomass by 69–98%, 63–88% and 41–74%, respectively, over positive control (2.42 mg) (**Table 3**; **Figure 3**). Likewise, different concentrations (10, 20, 40, 60, 80 and 100 mg mL^-1^) of the ethyl acetate, dichloromethane and *n-*hexane fractions of intracellular metabolites showed significant (p ≤ 0.05) reductions in fungal biomass by 48–85%, 42–70% and 35–62%, respectively, concerning the positive control (2.42 mg) (**Table 4**; **Figure 4**).

**Table 3:** Effect of different fractional concentrations of extracellular metabolites of *Bacillus subtilis* (BS-01) on the growth of *Alternaria solani* (AS).

Concentrations(mg mL^-1^)	Fungal biomass (mg)		
	*n*-Hexane	Dichloromethane	Ethyl acetate
+ve control (AS)	2.42 ± 0.133**^a^**	2.42 ± 0.133**^a^**	2.42 ± 0.146**^a^**
-ve control	2.23 ± 0.124**^a^**	1.89 ± 0.114**^ab^**	2.38 ± 0.167**^a^**
10 mg mL^-1^	1.79 ± 0.099**^b^**	1.81 ± 0.109**^b^**	1.62 ± 0.120**^b^**
20 mg mL^-1^	1.21 ± 0.061**^c^**	1.67 ± 0.117**^bc^**	0.99 ± 0.085**^c^**
40 mg mL^-1^	0.89 ± 0.048**^d^**	1.42 ± 0.093**^c^**	0.74 ± 0.077**^d^**
60 mg mL^-1^	0.38 ± 0.021**^e^**	1.11 ± 0.061**^d^**	0.26 ± 0.046**^e^**
80 mg mL^-1^	0.32 ± 0.019**^e^**	0.89 ± 0.054**^de^**	0.20 ± 0.04**^e^**
100 mg mL^-1^	0.28 ± 0.016**^e^**	0.64 ± 0.035**^e^**	0.04 ± 0.03**^f^**

Values with different superscript letters show a significant difference (p ≤ 0.05) in mean value of replicates (N=5) of each treatment as determined by LSD test. ± value indicates the standard error mean of replicates (N=5).

+ve control: with inoculation of *A. solani* (AS) only; -ve control: without inoculation of *A. solani* (AS) and applied organic solvent only.

**Table 4:** Effect of different fractional concentrations of intracellular metabolites of *Bacillus subtilis* (BS-01) on the growth of *Alternaria solani* (AS).

Concentrations (mg mL^-1^)	Fungal biomass (mg)		
	*n*-Hexane	Dichloromethane	Ethyl acetate
+ve control (AS)	2.42 ± 0.133**^a^**	2.42 ± 0.133**^a^**	2.42 ± 0.146**^a^**
0 mg mL^-1^ (-ve control)	2.23 ± 0.124**^a^**	1.89 ± 0.114**^ab^**	2.38 ± 0.167**^a^**
10 mg mL^-1^	1.79 ± 0.099**^b^**	1.81 ± 0.109**^b^**	1.62 ± 0.120**^b^**
20 mg mL^-1^	0.99 ± 0.061**^c^**	1.67 ± 0.117**^bc^**	1.21 ± 0.085**^bc^**
40 mg mL^-1^	0.74 ± 0.048**^d^**	1.42 ± 0.093**^c^**	0.89 ± 0.077**^c^**
60 mg mL^-1^	0.38 ± 0.021**^e^**	1.11 ± 0.061**^d^**	0.26 ± 0.046**^d^**
80 mg mL^-1^	0.32 ± 0.019**^e^**	0.89 ± 0.054**^de^**	0.20 ± 0.04**^d^**
100 mg mL^-1^	0.28 ± 0.016**^e^**	0.64 ± 0.035**^e^**	0.04 ± 0.03**^e^**

Values with different superscript letters show a significant difference (p ≤ 0.05) in mean value of replicates (N=3) of each treatment as determined by LSD test. ± value indicates the standard error mean of replicates (N=3).

+ve control: with inoculation of *A. solani* (AS) only; -ve control: without inoculation of *A. solani* (AS) and applied organic solvent only.

#### 3.3.1 GC-MS analysis of Extracellular metabolites

*B. subtilis* (LC425129.1.) provided an abundant source of biocidal compounds. All three fractions from extracellular metabolites were analyzed through GC-MS for the identification of the potential antifungal compounds. Based on peak area (%), these compounds were categorized into four groups i.e., most abundant, moderately abundant, less abundant and least abundant.

**Figure 5:**
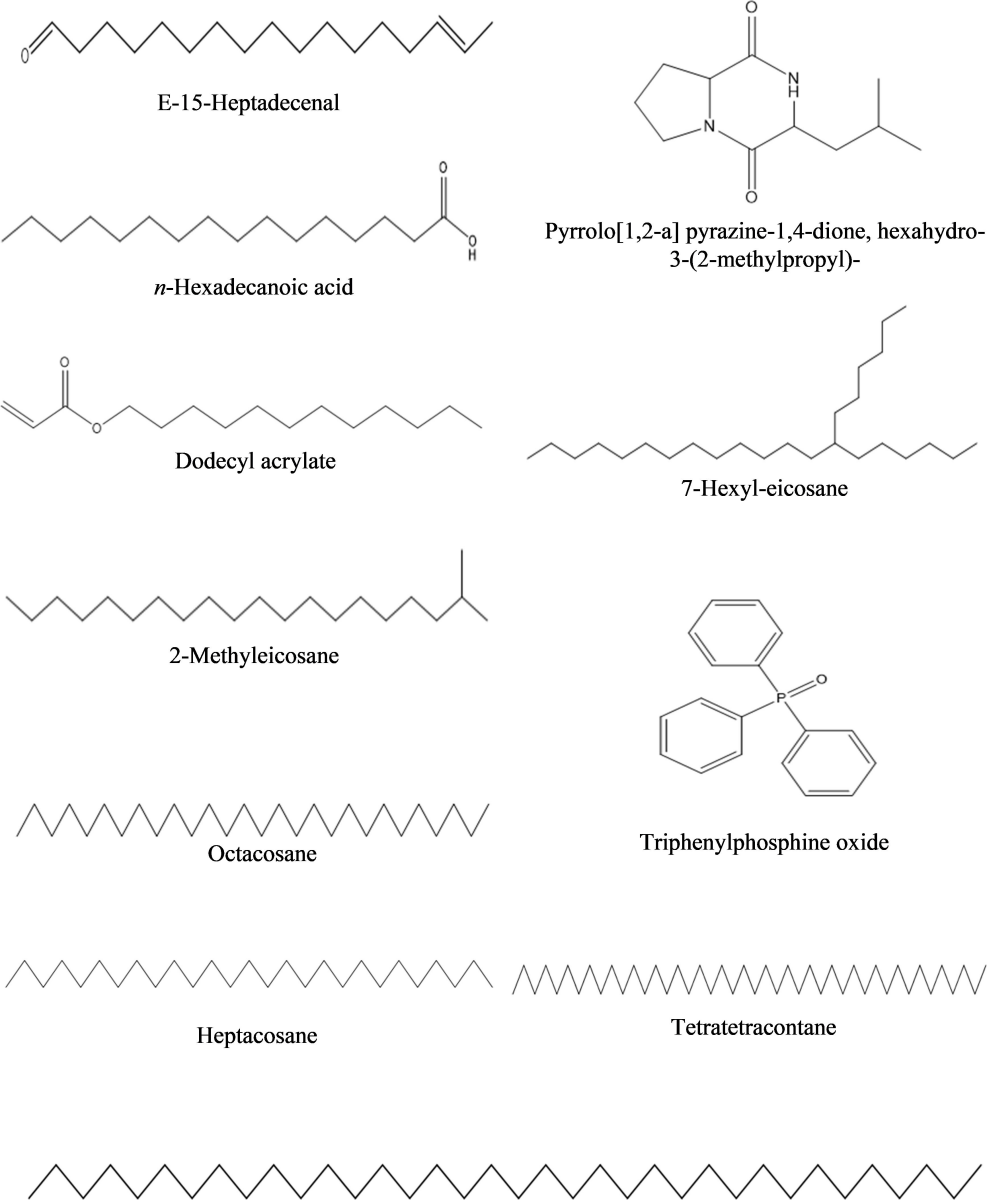
Structures of compounds identified from n-hexane fraction of extracellular metabolites of *Bacillus subtilis* (BS-01) through GC-MS analysis.

**a) *n*-Hexane fraction:** The GC-MS chromatogram analysis of the *n*-hexane fraction of bacterial extracellular metabolite specified eleven peaks (**Supplementary Figure S1**; **Table 5**). The most abundant compound was triphenylphosphine oxide (41.40%) followed by dodecyl acrylate (8.60%) as a moderately abundant compound. Five compounds [2-methyleicosane (3.0%); octacosane (3.0%); hexatriacontane (2.90%); *n*-hexadecanoic acid (2.40%); tetratetracontane (2.10%)] were found as less abundant compounds. The remaining four compounds viz., pyrrolo[1,2-a] pyrazine-1,4-dione, hexahydro-3-(2-methylpropyl)- (2.0%); 7-hexyleicosane (1.90%); heptacosane (1.40%) and E-15-heptadecenal (1.5%) were the least abundant compounds in the *n-*hexane fraction. The structures of these compounds are shown in **Figure 5**.

**Table 5:** Bioactive compounds identified from *n*-hexane fraction of extracellular metabolites of *Bacillus subtilis* (BS-01) through GC-MS analysis.

Sr. No.	Compounds	Molecular Formula	Molecular weight (g/mol)	Retention time(min)	Peakarea(%)
1	E-15-Heptadecenal	C_17_H_32_O	252	12.30	1.5
2	Pyrrolo[1,2-a] pyrazine-1,4-dione, hexahydro-3-(2-methylpropyl)-	C_11_H_18_N_2_O_2_	154	13.82	2.0
3	*n*-Hexadecanoic acid	C16H32O2	256	14.01	2.4
4	Dodecyl acrylate	C15H28O2	240	17.07	8.6
5	7-Hexyleicosane	C_26_H_54_	366	17.92	1.9
6	Tetratetracontane	C44H90	619	18.72	2.1
7	Triphenylphosphine oxide	C18H15OP	278	19.22	41.4
8	2-Methyleicosane	C21H44	296	19.49	3.0
9	Octacosane	C28H58	394	20.95	3.0
10	Hexatriacontane	C36H74	507	22.30	2.9
11	Heptacosane	C27H56	380	23.57	1.7

**Figure 6:**
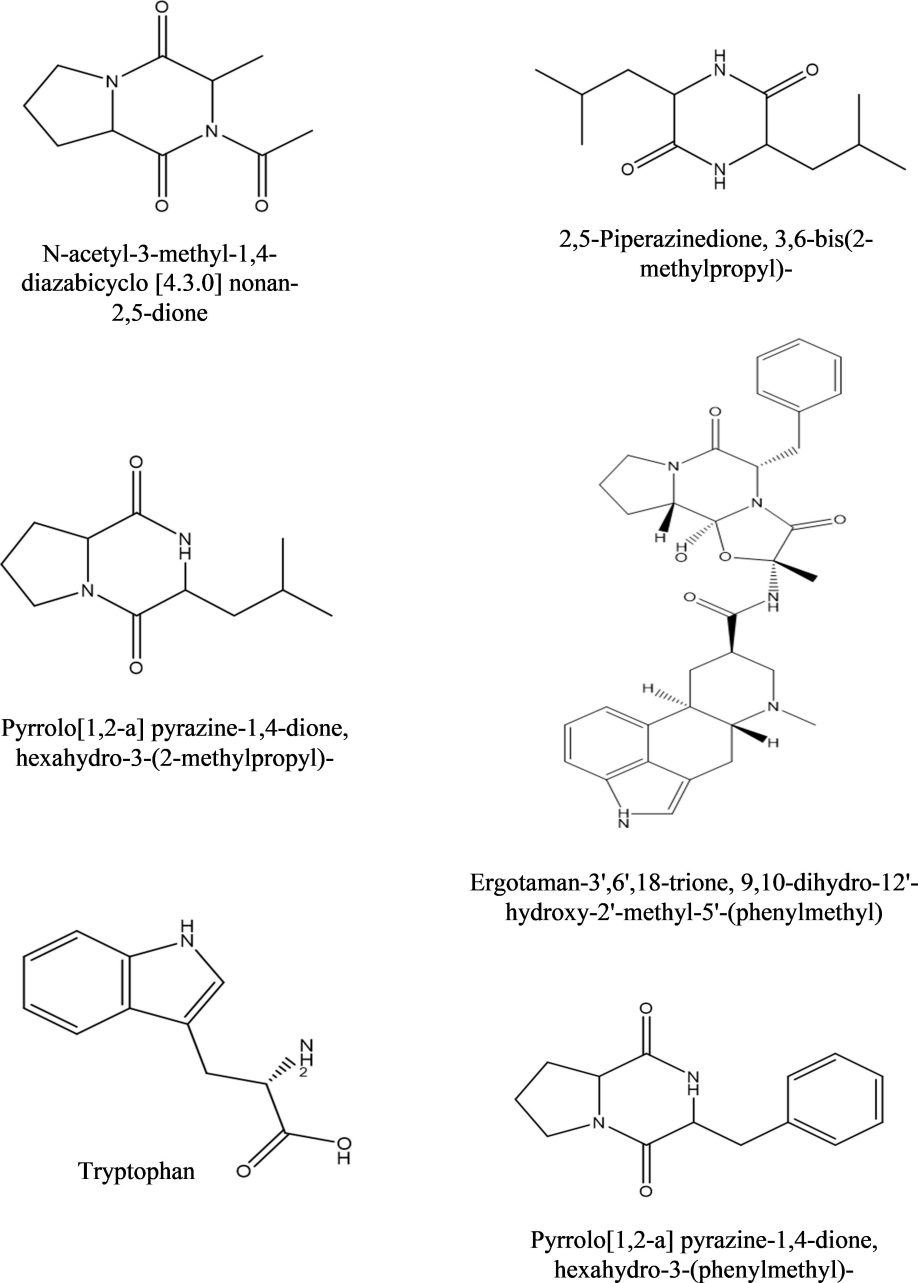
Structures of compounds identified from the dichloromethane fraction of extracellular metabolites of *Bacillus subtilis* (BS-01) through GC-MS analysis.

**b) Dichloromethane fraction:** GC-MS analysis revealed the occurrence of six compounds in the dichloromethane fraction of extracellular metabolites of BS-01 (**Supplementary Figure S2**; **Table 6**). Two compounds viz., pyrrolo[1,2-a] pyrazine-1,4-dione, hexahydro-3-(2-methylpropyl) and pyrrolo[1,2-a] pyrazine-1,4-dione, hexahydro-3-(phenylmethyl)- were categorized as the most abundant, exhibiting the highest proportion of 28.20% and 27.20%, respectively. Ergotaman-3’,6’,18-trione, 9,10-dihydro-12’-hydroxy-2’-methyl-5’-(phenylmethyl)-, (5’.alpha.,10.alpha.) was a moderately abundant compound (4.20%). Two compounds, including 3, 6-bis(2-methylpropyl)-2,5-piperazinedione (3.0%) and N-acetyl-3-methyl-1,4-diazabicyclo [4.3.0] nonan-2,5-dione (2.90%), were among the less abundant compounds and tryptophan (0.5%) occurred as the least abundant compound. The structures of these compounds are depicted in **Figure 6**.

**Table 6:** Bioactive compounds identified from dichloromethane fraction of extracellular metabolites of *Bacillus subtilis* (BS-01) through GC-MS analysis.

Sr. No.	Compounds	Molecular Formula	Molecular weight (g/mol)	Retention time (min)	Peak area (%)
1	N-acetyl-3-methyl-1,4-diazabicyclo [4.3.0] nonan-2,5-dione,	C_10_H_14_N_2_O_3_	210	11.60	2.9
2	Pyrrolo[1,2-a] pyrazine-1,4-dione, hexahydro-3-(2-methylpropyl)-	C_11_H_18_N_2_O_2_	210	14.03	28.2
3	2,5-Piperazinedione, 3,6-bis(2-methylpropyl)-	C_12_H_22_N_2_O_2_	226	14.07	3.0
4	Ergotaman-3’,6’,18-trione, 9,10-dihydro-12’-hydroxy-2’-Methyl-5’-(phenylmethyl)-, (5’.alpha.,10.alpha.)-	C_33_H_37_N_5_O_5_	583	17.72	4.2
5	Pyrrolo[1,2-a] pyrazine-1,4-dione, hexahydro-3-(phenylmethyl)-	C_14_H_16_N_2_O_2_	244	18.11	27.2
6	Tryptophan	C_11_H_12_N_2_O_2_	204	23.54	0.5

**Figure 7:**
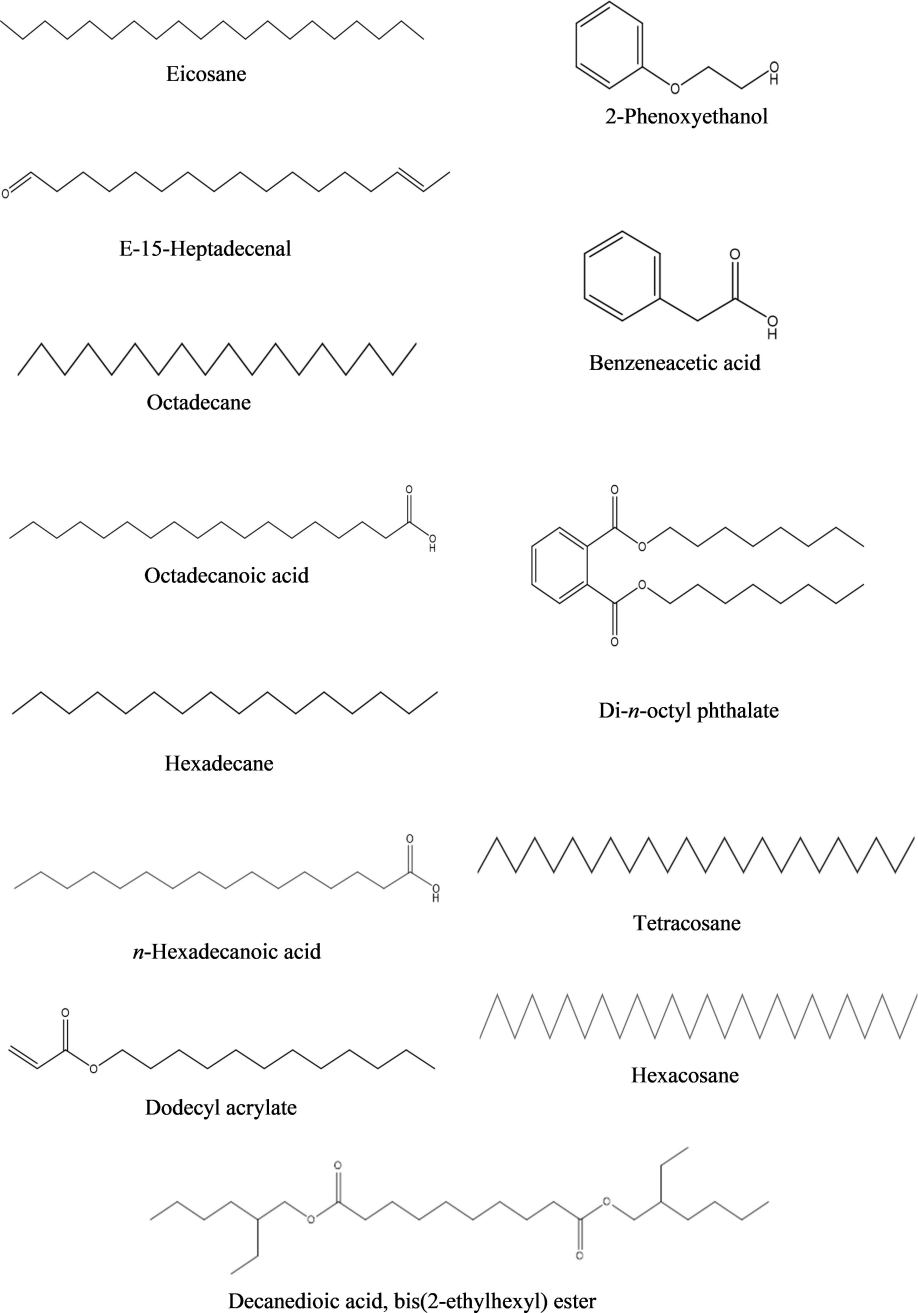
Structures of compounds identified from ethyl acetate fraction of extracellular metabolites of *Bacillus subtilis* (BS-01) through GC-MS analysis.

**c) Ethyl acetate fraction:** According to the GC-MS analysis, 13 bioactive compounds were identified in the ethyl acetate fraction of extracellular metabolites (**Supplementary Figure S3**; **Table 7**). Two compounds were documented as the most abundant [*n*-hexadecanoic acid (10.10%) and octadecane (7.10%)], while three compounds were moderately abundant [dodecyl acrylate (5.90%); eicosane (5.60%) and octadecanoic acid (5.60%)]. Tetracosane (4.78%) followed by benzeneacetic acid (3.60%) and di-*n*-octyl phthalate (2.40%) were less abundant compounds. Hexadecane (1.4%); decanedioic acid, bis(2-ethylhexyl) ester (1.20%); 2-phenoxyethanol (1.0%); E-15-heptadecenal (1.0%) and hexacosane (0.70%) were the least abundant compounds. The structures of these compounds are presented in **Figure 7**.

**Table 7:** Bioactive compounds identified from ethyl acetate fraction of extracellular metabolites of *Bacillus subtilis* (BS-01) through GC-MS analysis.

Sr. No.	Compounds	Molecular Formula	Molecular weight (g/mol)	Retention time(min)	Peakarea(%)
1	2-Phenoxyethanol	C8H10O2	138	5.59	1.0
2	Benzeneacetic acid	C8H8O2	136	5.95	3.6
3	Hexadecane	C16H34	226	7.73	1.4
4	Eicosane	C20H42	282	10.12	5.6
5	Dodecyl acrylate	C15H28O2	240	11.21	5.9
6	E-15-Heptadecenal	C17H32O	252	12.29	1.0
7	Octadecane	C18H38	254	12.37	7.1
8	n-Hexadecanoic acid	C16H32O2	256	14.06	10.1
9	Tetracosane	C24H50	338	14.38	4.78
10	Octadecanoic acid	C18H36O2	284	15.93	5.6
11	Di-n-octyl phthalate	C24H38O4	390	19.17	2.4
12	Hexacosane	C26H54	366	19.48	0.7
13	Decanedioic acid, bis(2-ethylhexyl) ester	C26H50O4	426	21.03	1.2

#### 3.3.2 GC-MS analysis of Intracellular metabolites

The identification of the potential antifungal compounds exhibited in all three fractions from intracellular metabolites of BS-01 were analyzed through GC-MS as mentioned above.

**Figure 8:**
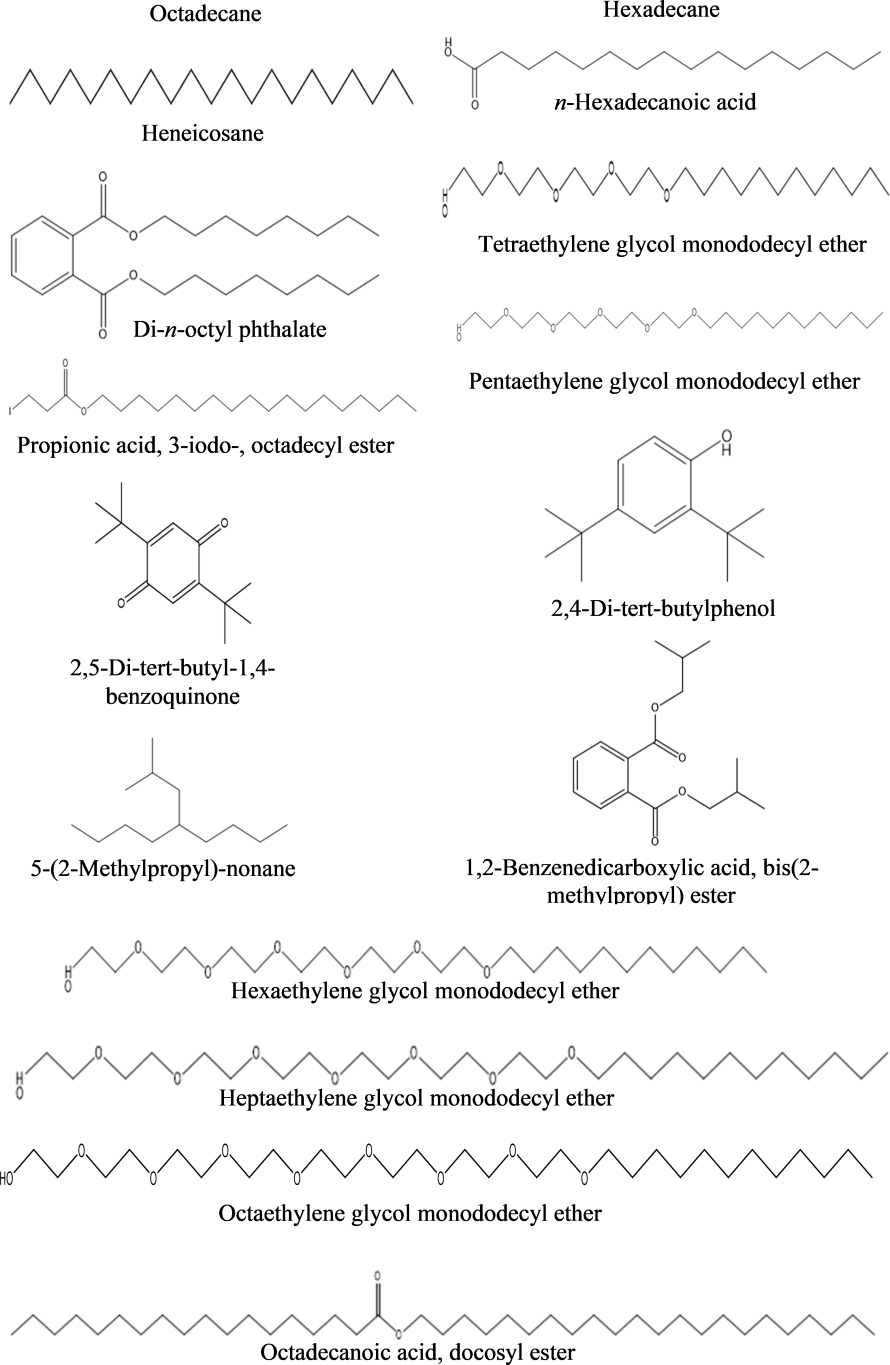
Structures of compounds identified from the n-hexane fraction of intracellular metabolites of *Bacillus subtilis* (BS-01) through GC-MS analysis.

**a) *n-*Hexane fraction:** The GC-MS chromatogram revealed 16 peaks from the *n-*hexane fraction of intracellular metabolites of BS-01 (**Supplementary Figure S4**; **Table 8**). The chromatogram revealed the occurrence of octaethylene glycol monododecyl ether (1.95%); pentaethylene glycol monododecyl ether (1.90%) and hexaethylene glycol monododecyl ether (1.77%) at the highest frequency (most abundant). However, four other compounds viz., di-*n*-octyl phthalate (1.35%); heptaethylene glycol monododecyl ether (1.07%); *n*-hexadecanoic acid (1.04%) and propionic acid, 3-iodo-, octadecyl ester (1.04%) were observed in lower amounts (moderately abundant). Four compounds viz., 5-(2-methylpropyl)-nonane; octadecanoic acid; heneicosane and tetraethylene glycol monododecyl ether, displayed as less abundant compounds, while the remaining five compounds [octadecane; 1,2-benzenedicarboxylic acid, bis(2-methylpropyl) ester; hexadecane; 2,4-di-terta-butylphenol and 2,5-di-terta-butyl-1,4-benzoquinone exhibited less than 0.5% abundance and ranked as least abundant. The structures of these compounds are displayed in **Figure 8**.

**Table 8:** Bioactive compounds identified from *n*-hexane fraction of intracellular metabolites of *Bacillus subtilis* (BS-01) through GC-MS analysis.

Sr. No.	Compounds	Molecular Formula	Molecular weight (g/mol)	Retention time (min)		Peak area(%)	
1	Octadecanoic acid	C_40_H_80_O_2_	593	27.2		0.96
2	Heptaethylene glycol monododecyl ether	C_26_H_54_O_8_	494	25.7		1.07
3	Hexaethylene glycol monododecyl ether	C_24_H_50_O_7_	450	25.0		1.77
4	Octaethylene glycol monododecyl ether	C_28_H_58_O_9_	538	22.3		1.95
5	Pentaethylene glycol monododecyl ether	C_22_H_46_O_6_	406	21.6		1.9
6	Heneicosane	C_21_H_44_	296	17.9		0.59
7	Di-*n*-octyl phthalate	C_24_H_38_O_4_	390	19.1		1.35
8	Tetraethylene glycol monododecyl ether	C_20_H_42_O_5_	362	18.3		0.66
9	Propionic acid, 3-iodo-, octadecyl ester	C_21_H_41_IO_2_	452	17.0		1.04
10	Octadecane	C_18_H_38_	254	14.4		0.43
11	*n*-Hexadecanoic acid	C_16_H_32_O_2_	256	13.9		1.04
12	1,2-Benzenedicarboxylic acid, bis(2-methylpropyl) ester	C_16_H_22_O_4_	278	13.1		0.13
13	Hexadecane	C_16_H_34_	226	10.1		0.06
14	2,4-Di-tert-butylphenol	C_14_H_22_O	206	9.1		0.02
15	2,5-Di-tert-butyl-1,4-benzoquinone	C_14_H_20_O_2_	220	8.7		0.02
16	5-(2-Methylpropyl)-nonane	C_13_H_28_	184	6.2		0.96

**Figure 9:**
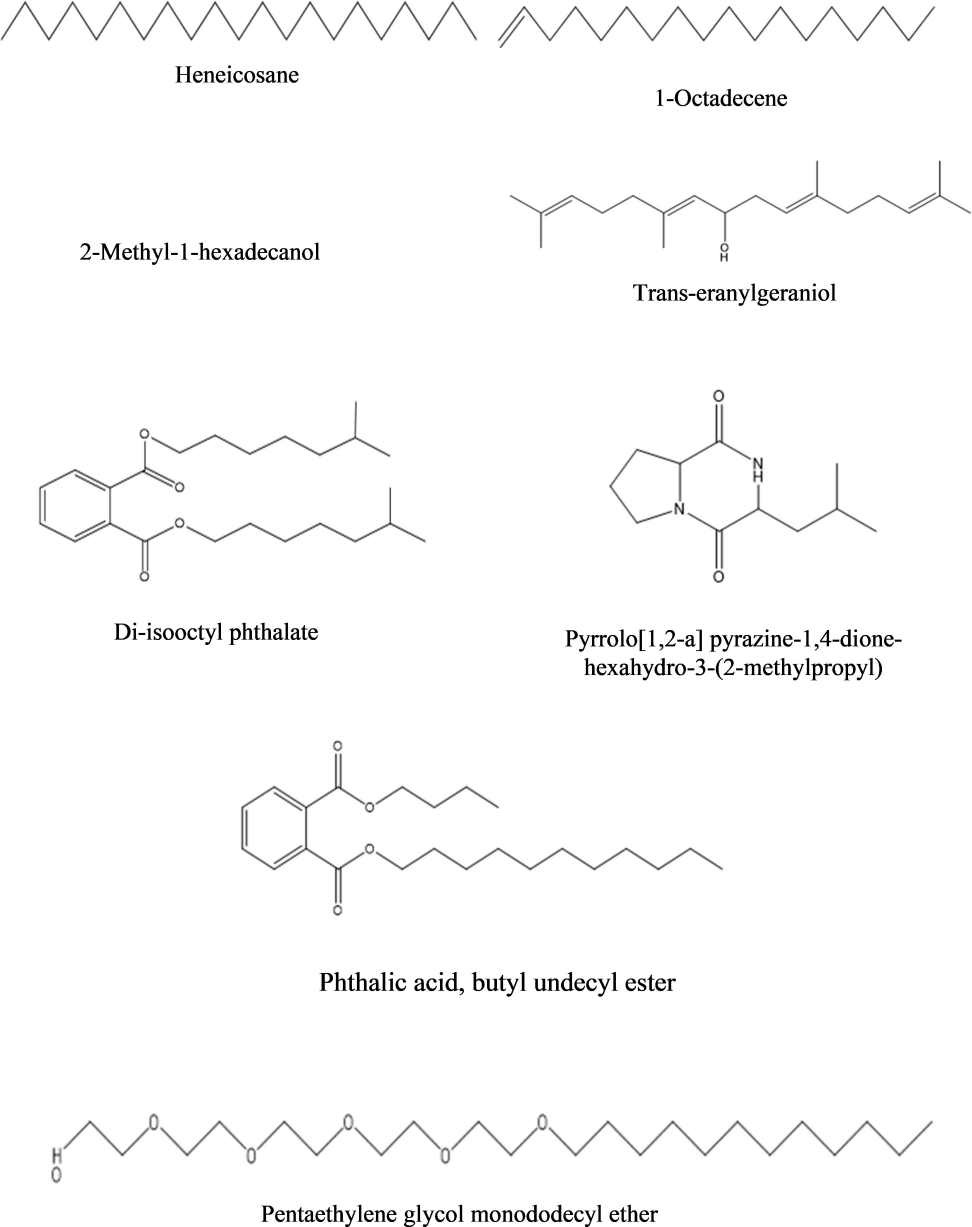
Structures of compounds identified from the dichloromethane fraction of intracellular metabolites of *Bacillus subtilis* (BS-01) through GC-MS analysis.

**b) Dichloromethane fraction:** The GC-MS analysis of the dichloromethane fraction of intracellular metabolites of BS-01 detected eight compounds (**Supplementary Figure S5**; **Table 9**). Only one compound was noticed as the most abundant compound [phthalic acid, butyl undecyl ester (1.07%)]. Trans-geranylgeraniol (0.67%) and heneicosane (0.63%) were among the moderately abundant compounds, while pyrrolo[1,2-a] pyrazine-1,4-dione, hexahydro-3-(2-methylpropyl)- (0.55%); 1-octadecene (0.55%) and 2-methyl-1-hexadecanol (0.45%) were found as less abundant compounds. Pentaethylene glycol monododecyl ether and disooctyl phthalate were detected in a similar proportion and classified as the least abundant compounds (0.34%). The structures of these compounds are presented in **Figure 9**.

**Table 9:** Bioactive compounds identified from dichloromethane fraction of intracellular metabolites of *Bacillus subtilis* (BS-01) through GC-MS analysis.

Sr. No.	Compounds	Molecular Formula	Molecular weight (g/mol)	Retention time(min)	Peak area(%)
1	Trans-geranylgeraniol	C_20_H_34_O	290	24.8	0.67
2	Heneicosane	C_21_H_44_	296	19.4	0.62
3	Pentaethylene glycol monododecyl ether	C_22_H_46_O_6_	406	19.7	0.34
4	Diisooctyl phthalate	C_24_H_38_O_4_	390	19.1	0.34
5	Pyrrolo[1,2-a] pyrazine-1,4-dione, hexahydro-3-(2-methylpropyl)-	C_11_H_18_N_2_O_2_	210	13.8	0.55
6	Phthalic acid, butyl undecyl ester	C_23_H_36_O_4_	376	13.1	1.07
7	2-Methyl-1-hexadecanol	C_17_H_36_O	256	11.7	0.45
8	1-Octadecene	C_18_H_36_	252	11.5	0.55

**Figure 10:**
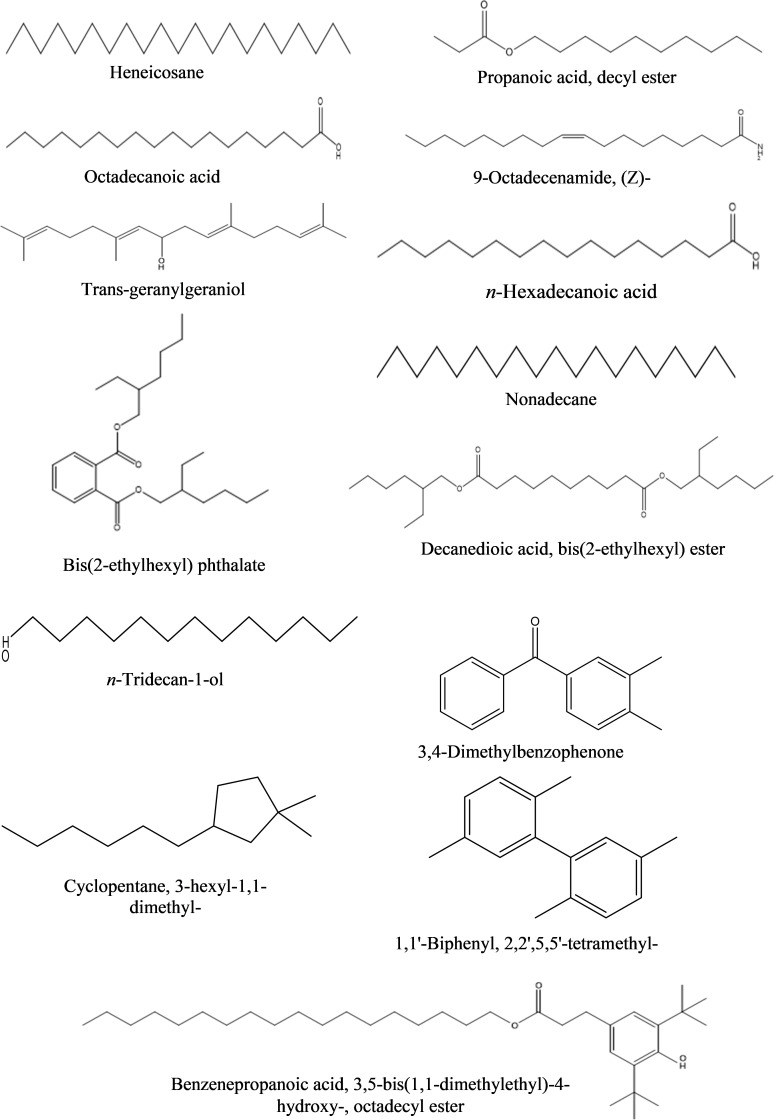
Structures of compounds identified from the ethyl acetate fraction of intracellular metabolites of *Bacillus subtilis* (BS-01) through GC-MS analysis.

**c) Ethyl acetate fraction:** The GC-MS analysis of ethyl acetate fraction of intracellular metabolite resulted in the identification of 14 compounds (**Supplementary Figure S6**; **Table 10**). The most abundant compounds identified were *n*-hexadecanoic acid (7.73%) and *n*-tridecan-1-ol (6.15%). Moderately abundant compounds were octadecanoic acid (5.04%) and cyclopentane, 3-hexyl-1,1-dimethyl- (4.13%). Whilst, bis(2-ethylhexyl) phthalate (2.15%); nonadecane (1.97%); 1,1’-biphenyl, 2,2’,5,5’-tetramethyl- (1.46%); 3,4-dimethylbenzophenone (1.03%) and heneicosane (1.01%) were ranked as less abundant compounds. In addition, the remaining compounds [benzenepropanoic acid 3,5-bis(1,1-dimethylethyl)-4-hydroxy-, octadecyl ester; trans-geranylgeraniol; decanedioic acid, bis(2-ethylhexyl) ester; 9-octadecenamide, (Z)- and propanoic acid, decyl ester] were present in the range of 0.99–0.35%. The structures of these compounds are displayed in **Figure 10**.

**Table 10:** Bioactive compounds identified from ethyl acetate fraction of intracellular metabolites of *Bacillus subtilis* (BS-01) through GC-MS analysis.

Sr. No.	Compounds	Molecular Formula	Molecular weight (g/mol)	Retention time(min)	Peak area(%)
1	Benzenepropanoic acid, 3,5-bis(1,1-dimethylethyl)-4-hydroxy-, octadecyl ester	C_35_H_62_O_3_	530	26.1	0.98
2	Trans-geranylgeraniol	C_20_H_34_O	290	24.8	0.46
3	Decanedioic acid, bis(2-ethylhexyl) ester	C_26_H_50_O_4_	426	20.9	0.77
4	Bis(2-ethylhexyl) phthalate	C_24_H_38_O_4_	390	19.1	2.15
5	9-Octadecenamide, (Z)-	C_18_H_35_NO	281	17.6	0.37
6	Propanoic acid, decyl ester	C_13_H_26_O_2_	214	17.1	0.35
7	Heneicosane	C_21_H_44_	296	16.2	1.01
8	Octadecanoic acid	C_18_H_36_O_2_	284	15.9	5.04
9	Nonadecane	C_19_H_40_	268	14.3	1.97
10	*n*-Hexadecanoic acid	C_16_H_32_O_2_	256	14.0	7.73
11	3,4-Dimethylbenzophenone	C_15_H_14_O	210	13.5	1.03
12	Cyclopentane, 3-hexyl-1,1-dimethyl-	C_13_H_26_	182	11.9	4.13
13	1,1’-Biphenyl, 2,2’,5,5’-tetramethyl-	C_34_H_36_	444	11.5	1.46
14	*n*-Tridecan-1-ol	C_13_H_28_O	200	11.2	6.15

## 4 Discussion

A bacterial strain named “BS-01” was characterized to divulge its identity due to its clear antifungal effect against *A. solani* and its 16rDNA sequence displayed >99.5% homology with the *Bacillus subtilis*. Our results indicated that strain BS-01 is closely related to numerous species by exhibiting high similarities (≥ 99%). Several strains of *B. subtilis* and some other species of the genus are often found as colonizers of the internal tissues of plants and promote plant growth even under stress (Han et al., 2015; Abbas et al. (2014)). To assess the impact of preventive and curative treatments of *B. subtilis* (BS-01) on the pathogen load in *A. solani*-infected tomato plants, BS-01 was applied before or after the pathogen’s inoculation. The tomato plants or seeds inoculated with BS-01 before infection of *A. solani* (preventive) exhibited a significantly lower pathogen load as compared to the BS-01 application on plants or in soil, after pathogen challenge (curative). This observation corresponds to results found by El-Sheikh et al. (2002), who stated that protective treatments with antagonistic *Bacillus* spp. were more effective than curative treatments to control *Phytophthora infestans* in potato crops. Ongena et al. (2005) also suggested that pre-treatment of BS-01 not only produces bioactive compounds but also sensitized tomato plants to subsequently reduce a pathogen infestation. Plant protection as conferred by bacteria (*B. subtilis*) used in this study could result from the induction of systemic resistance which enhances biological control over tomato early blight through direct antagonisms (Khanna et al., 2019b; Awan et al., 2022). The antifungal activity of this strain BS-01 against *A. solan* has been tested in a previous study (Shoaib et al., 2019). In another work, Awan et al. (2022) also studied tomato early blight is significantly managed with the application of a biocontrol agent namely *Bacillus subtilis* (BS-01) along with plant nutrients in the field.

In addition, the current study investigated the antifungal assays with the various concentrations (10-100 mg mL^-1^) of extra- and intracellular bacterial metabolites of BS-01 extracted in organic solvents (*n*-hexane, dichloromethane, and ethyl acetate) revealed that higher concentrations (>40 mg mL^-1^) of different fractions showed a noticeable antifungal effect against *A. solani* (Farhana et al., 2014). Results of our GC-MS analyses also showed an abundance of compounds of antifungal origin in all three organic fractions of both extra- and intracellular metabolites. The ethyl acetate fraction from both extra- and intracellular metabolites showed a strong inhibition in fungal biomass (69–98% and 48–85%) followed by *n*-hexane (63–88% and 35–62%) and dichloromethane (41–74% and 42–70%), respectively, indicating that the percentage of the bioactive compounds in the ethyl acetate fraction after each step of purification has increased. Therefore, it exhibited antifungal activity at a lower concentration than those for the other fractions (Al-Saraireh et al., 2015). Former studies on *Bacillus* metabolites extracted in organic solvents revealed that ethyl acetate and chloroform fractions of *Bacillus* strains hold greater antifungal potential due to chemical multiplicity (peptide, polyketide, lipopeptide, phospholipid, and others) (Numan et al., 2022).

The extracellular fraction of ethyl acetate, *n*-hexane and dichloromethane displayed the presence of 13, 11 and 6 biocidal compounds, respectively. However, the most abundant compound identified in the ethyl acetate fraction of extracellular metabolite was *n*-hexadecanoic acid (10.10%) and octadecane (7.10%) followed by three moderately abundant compounds viz., dodecyl acrylate, eicosane and octadecanoic acid in the range of 5.6–5.9%. Such compounds were also identified by Bharose and Gajera (2018) from a crude extract of *Bacillus* and *Pseudomonas* metabolites. *n*-hexadecanoic acid (fatty acid) is a potential antifungal, antibacterial, antioxidant, anticancer, nematicide and pesticide compound which has been isolated from many medicinal plants (Umaiyambigai et al., 2017) and metabolites of *B. subtilis* strain HD16b. Octadecane (alkane) identified from volatile organic compounds of *Bacillus pumilu* displayed the strongest inhibition against *Penicillium italicum* (Morita et al., 2019). Eicosane (an alkane) has antioxidant, antimicrobial and antifungal properties (Theng and Korpenwar, 2015) and has been used against target spots in tobacco leaf caused by *Rhizoctonia solani* (Ahsan et al., 2017). Likewise, octadecanoic acid (ethyl ester) isolated with other compounds from *Bacillus atrophaeus* strain showed the potential to manage *Verticillium* wilt (Mohamad et al., 2018). Another important compound i.e. tetracosane (alkane) was recorded as less abundant (4.78%) in the ethyl acetate fraction, however, it is also used as an antibacterial, antifungal and anticancer compound. Ni et al. (2018) documented a strong inhibitory effect of *Bacillus atrophaeus* against *Botrytis cinerea* and suggested the presence of tetracosane with other compounds (octadecanoic acid, hexadecane, 2-methyl- and eicosane, etc.) in their dichloromethane fraction of bacterial metabolites.

In the *n-*hexane fraction of extracellular metabolites of BS-01, triphenylphosphine oxide (TPPO: 41.40%) was detected as the most abundant compound followed by dodecyl acrylate (8.60%) as a moderately abundant compound. TPPO is a popular organophosphorus compound, which has been extensively employed as a ligand for many metals and the resulting compounds indicated strong antimicrobial activities (Karakus et al., 2014; Faiz et al., 2016). TPPO exhibited potential antifungal activities due to mitochondrial dysfunction in *Candida albicans* as TPP^+^-conjugates can bypass active expulsion by efflux pumps and accumulate in the fungal mitochondria to exert fungicidal activity (Chang et al., 2018). Dodecyl acrylate (ester), isolated from secondary metabolites of *Streptomyces werraens* has also shown antifungal potential against *Fusarium oxysporum* (Singh and Wahla, 2018). Bharose and Gajera (2018) reported that dodecyl acrylate extracted from *B. subtilis* has strong antioxidant and antifungal effects against the aflatoxin-producing fungus *Aspergillus flavus*.

The most abundant compound detected from the dichloromethane fraction of extracellular metabolites was pyrrolo[1,2-a] pyrazine-1,4-dione, hexahydro-3-(2-methylpropyl) (28.20%) and a moderately abundant compound was pyrrolo[1,2-a] pyrazine-1,4-dione, hexahydro-3-(phenylmethyl)- (27.20%). Likewise, both of these organic compounds have been reported as possible antifungal compounds from *Bacillus* and *Stre*p*tomyces* species (Manimaran et al., 2017). Kiran et al. (2018) and Tangjitjaroenkun (2018) reported that pyrrolo[1,2-a] pyrazine-1,4-dione, hexahydro-3-(2-methylpropyl)- is a strong antioxidant agent isolated from *Bacillus* and *Streptomyces* spp., exhibiting antimicrobial and antifungal activity against various pathogenic bacteria (*Staphylococcus aureus, Enterobacter cloacae, Klebsiella pneumoniae*, and *Bacillus subtilis*) and fungi (*Pyricularia oryzae*).

However, GC-MS profiles of intracellular metabolites of ethyl acetate, dichloromethane and *n*-hexane fractions exhibited the occurrence of 14, 8 and 16 bioactive compounds, respectively. In the ethyl acetate fraction, *n*-hexadecanoic acid (7.73%) and *n*-tridecan-1-ol (6.15%) were the most abundant, while octadecanoic acid (5.04%) was among the moderately abundant compounds. *n*-tridecan-1-ol (alkane) is used in the production of detergents and surfactants, cosmetics, foods, industrial solvents as an effective antimicrobial compound (Garaniya and Bapodra, 2014). Ojinnaka et al. (2016) detected octadecanoic acid from the crude extracts of *Buchholzia coriacea* and showed its bioactivity against several fungi and bacteria.

In the dichloromethane fraction of intracellular metabolites from BS-01, the most abundant compounds were phthalic acid, butyl undecyl ester (1.07%), while trans-geranylgeraniol (0.67%) and heneicosane (0.63%) were among the moderately abundant compounds. Phthalic acid (dicarboxylic acid) is a benzoic acid derivative and is known for its antimycotic potential. Lago et al. (2004) documented fungitoxic activity of benzoic acid derivatives isolated from P*iper* species against phytopathogenic fungi i.e. *Cladosporium cladosporioides* and *Clados*p*orium sphaerospermum*. Trans-geranylgeraniol is present in medicinal plants (*Bauhinia variegata* and *Garcinia cambogia*) and exhibited pharmaceutical value.

The *n-*hexane fraction of intracellular metabolites contained octaethylene glycol monododecyl ether (1.95%); pentaethylene glycol monododecyl ether (1.90%) and hexaethylene glycol monododecyl ether (1.77%) as the most abundant compounds. These alcoholic compounds have been reported to show variable antimicrobial activity. For example, octaethylene glycol monododecyl ether acts as detergent and displayed antimicrobial potential (Nardello-Rataj and Leclercq, 2014); pentaethylene glycol monododecyl ether is a surfactant used to reduce the development of powdery mildew on cucumber plants (Yu et al., 2009), and hexaethylene glycol monododecyl ether is also a surfactant and reportedly acts as anti-microbial and antifungal compound (Angarska et al., 2015). Nardello-Rataj and Leclercq (2016) also revealed that a mixture of two surfactants viz, octaethylene glycol monododecyl ether and didecyldimethylammonium chloride, showed a high synergistic effect against enveloped viruses. Our results indicated that biocidal compounds identified in the present study from different fractions of extra- and intracellular metabolites of BS-01 belonging to long chains of alkanes, fatty acids, esters, and alkyl polyglycol ethers, which might have significant antifungal properties against *A. solani* as confirmed *in vitro* antifungal assays.

## 5 Conclusion

Controlling fungal growth (*A. solani*) and early blight severity in tomato plants by employing the biocontrol agent *Bacillus subtilis* (BS-01) is a safe, effective and sustainable approach in contrast to synthetic pesticides. The strain was identified and characterized based on 16S rDNA sequence analysis. Our results indicated that *Bacillus subtilis* (BS-01) produces potent bioactive VOCs extracted from extra- and intra-cellular metabolites which are effective in inhibiting the growth of *Alternaria solani*. Afterward, the active constituents (VOCs) of this strain (BS-01) which could have a potential antifungal impact were identified by GC–MS. Besides, it was studied that preventive measure to control the pathogenic attack is more sound to reduce the pathogenic load on tomato foliage than a curative measure against tomato early blight. The current results suggest that the application of *B. subtilis* as a potential biocontrol agent not only enables the production of bioactive compounds but may suppress *A. solani*-associated diseases and could potentially be applied in multiple horticultural crops.

## Data availability statement

The datasets presented in this study can be found in online repositories. The names of the repository/repositories and accession number(s) can be found in the article/**Supplementary Material**.

## Author contributions

ZAA prepared the draft. All the authors mentioned in the manuscript have made a substantial, direct, and intellectual contribution to the work and have approved it for publication.

## References

[B1] Abbas M. T. Hamza M. A. Youssef H. H. Youssef G. H. Fayez M. Monib M.. (2014). Bio-preparates support the productivity of potato plants grown under desert farming conditions of north Sinai: Five years of field trials. J. Advan. Res. 5 (1), 41–48. doi: 10.1016/j.jare.2012.11.004 25685470 PMC4294711

[B2] Ahsan T. Chen J. Zhao X. Irfan M. Wu Y. (2017). Extraction and identification of bioactive compounds (eicosane and dibutyl phthalate) produced by *Streptomyces* strain KX852460 for the biological control of *Rhizoctonia solani* AG-3 strain KX852461 to control target spot disease in tobacco leaf. AMB Express 7 (1), 1–9. doi: 10.1186/s13568-017-0351-z 28255861 PMC5334330

[B3] Al-Saraireh H. Al-Zereini W. A. Tarawneh K. A. (2015). Antimicrobial activity of secondary metabolites from a soil *Bacillus* sp. 7B1 isolated from south Al-karak, jordan. Jordan. J. Biol. Sci. 8, 127–132. doi: 10.12816/0027558

[B4] Angarska J. K. Ivanova D. S. Manev E. D. (2015). Drainage of foam films stabilized by nonionic, ionic surfactants and their mixtures. colloids surfaces a physicochem. Eng. Asp. 481, 87–99. doi: 10.1016/j.colsurfa.2015.04.043

[B5] Awan Z. A. Shoaib A. Khan K. A.. (2018). Variations in total phenolics and antioxidant enzymes cause phenotypic variability and differential resistant response in tomato genotypes against early blight disease. Sci. Hortic. 239, 216–223. doi: 10.1016/j.scienta.2018.05.044

[B6] Awan Z. A. Shoaib A. (2019). Combating early blight infection by employing *Bacillus subtilis* in combination with plant fertilizers. Curr. Plant Biol. 20, 100125. doi: 10.1016/j.cpb.2019.100125

[B7] Awan Z. A. Shoaib A. Iftikhar M. S. Jan B. L. Ahmad P. (2022). Combining biocontrol agent with plant nutrients for integrated control of tomato early blight through the modulation of physio-chemical attributes and key antioxidants. Front. Microbiol. 13. doi: 10.3389/fmicb.2022.807699 PMC8986128 35401436

[B8] Bharose A. Gajera H. (2018). Antifungal activity and metabolites study of bacillus strain against aflatoxin producing aspergillus. J. Appl. Microbiol. Biochem. 2 (8), 1–8. doi: 10.21767/2576-1412.100024

[B9] Chandrasekaran M. Chun S. C. (2016). Expression of PR-protein genes and induction of defense-related enzymes by Bacillus subtilis CBR05 in tomato (Solanum lycopersicum) plants challenged with Erwinia carotovora subsp. carotovora. Biosci. Biotech. and Biochem. 80, 2277–2283. 10.1080/09168451.2016.1206811 27405462

[B10] Chang W. Liu J. Zhang M. Shi H. Zheng S. Jin X.. (2018). Efflux pump-mediated resistance to antifungal compounds can be prevented by conjugation with triphenylphosphonium cation. Nat. Commun. 9, 5102. doi: 10.1038/s41467-018-07633-9 30504815 PMC6269435

[B11] Daranas N. Roselló G. Cabrefiga J. Donati I. Francés J. Badosa E.. (2019). Biological control of bacterial plant diseases with lactobacillus plantarum strains selected for their broad-spectrum activity. Ann. Appl. Biol. 174, 92–105. doi: 10.1111/aab.12476 30686827 PMC6334523

[B12] Faiz Y. Zhao W. Feng J. Sun C. He H. Zhu J. (2016). Occurrence of triphenylphosphine oxide and other organophosphorus compounds in indoor air and settled dust of an institute building. Build. Environ. 106, 196–204. doi: 10.1016/j.buildenv.2016.06.022

[B13] Farhana S. Ab S. Sijam K. Omar D. (2014). Chemical composition of piper sarmentosum extracts and antibacterial activity against the plant pathogenic bacteria *Pseudomonas fuscovaginae* and *Xanthomonas oryzae* pv . oryzae. J. Plant Dis. Prot. 121, 237–242. doi: 10.1007/BF03356518

[B14] Garaniya N. Bapodra A. (2014). Ethno botanical and phytophrmacological potential of abrus precatorius l.: A review. Asian Pac. J. Trop. Biomed. 4, S27–S34. doi: 10.12980/APJTB.4.2014C1069 25183095 PMC4025349

[B15] Gouda S. Kerry R. G. Das G. Paramithiotis S. Shin H. S. Patra J. K. (2018). Revitalization of plant growth promoting rhizobacteria for sustainable development in agriculture. Microbiol. Res. doi: 10.1016/j.micres.2017.08.016 29146250

[B16] Guo Q. Dong W. Li S. Lu X. Wang P. Zhang X.. (2014). Fengycin produced by *Bacillus subtilis* NCD-2 plays a major role in biocontrol of cotton seedling damping-off disease. Microbiol. Res. 169, 533–540. doi: 10.1016/j.micres.2013.12.001 24380713

[B17] Hadi A.. (2013). A critical appraisal of Grice’s cooperative principle. Open J Mod. Ling. 3, 69–72. doi: 10.4236/ojml.2013.31008

[B18] Han Q. Wu F. Wang X. Qi H. Shi L. Ren A.. (2015). The bacterial lipopeptide iturins induce *Verticillium dahliae* cell death by affecting fungal signalling pathways and mediate plant defence responses involved in pathogen-associated molecular pattern-triggered immunity. Environ. Microbiol. 17, 1166–1188. doi: 10.1111/1462-2920.12538 24934960

[B19] Hashem A. Tabassum B. Fathi Abd_Allah E. (2019). *Bacillus subtilis*: A plant-growth promoting rhizobacterium that also impacts biotic stress. Saudi J. Biol. Sci. 26, 1219-1297. doi: 10.1016/j.sjbs.2019.05.004 PMC6734152 31516360

[B20] Hadimani B. R. Kulkarni S. (2016). Bioefficacy of B diseases of tomato Bacillus subtilis against foliar fungal diseases of tomato. Int. J. Appl. Pure Sci. Agric. 3 (2), 220–27.

[B21] Hussain A. Ahmad M. Nafees M. Iqbal Z. Luqman M. Jamil M.. (2020). Plant-growth-promoting bacillus and *Paenibacillus* species improve the nutritional status of *Triticum aestivum* l. PloS One 15 (12), e0241130. doi: 10.1371/journal.pone.0241130 33259487 PMC7707572

[B22] Ilyas N. Akhtar N. Yasmin H. Sahreen S. Hasnain Z. Kaushik P.. (2022). Efficacy of citric acid chelate and bacillus sp. in amelioration of cadmium and chromium toxicity in wheat. Chemosphere 290, 133342. doi: 10.1016/j.chemosphere.2021.133342 34922965

[B23] Karakus M. Ikiz Y. Kaya H. I. Simsek O. (2014). Synthesis, characterization, electrospinning and antibacterial studies on triphenylphosphine-dithiphosphonates Copper(I) and Silver(I) complexes. Chem. Cent. J. doi: 10.1186/1752-153X-8-18 PMC4004045 24629061

[B24] Khanna K. Jamwal V. L. Kohli S. K. Gandhi S. G. Ohri P. Bhardwaj R.. (2019b). Role of plant growth promoting bacteria (PGPRs) as biocontrol agents of meloidogyne incognita through improved plant defense of *Lycopersicon esculentum* . Plant Soil 436 (1), 25–345. doi: 10.1007/s11104-019-03932-2

[B25] Khanna K. Jamwal V. L. Sharma A. Gandhi S. G. Ohri P. Bhardwaj R.. (2019a). Supplementation with plant growth promoting rhizobacteria (PGPR) alleviates cadmium toxicity in *Solanum lycopersicum* by modulating the expression of secondary metabolites. Chemosphere 230, 628–639. doi: 10.1016/j.chemosphere.2019.05.072 31128509

[B26] Kiran G. S. Priyadharsini S. Sajayan A. Ravindran A. Selvin J. (2018). An antibiotic agent pyrrolo[1,2-: A] pyrazine-1,4-dione,hexahydro isolated from a marine bacteria *Bacillus tequilensis* MSI45 effectively controls multi-drug resistant *Staphylococcus aureus* . RSC Adv. 8, 17837–17846. doi: 10.1039/c8ra00820e 35542054 PMC9080480

[B27] Köhl J. Kolnaar R. Ravensberg W. J. (2019). Mode of action of microbial biological control agents against plant diseases: Relevance beyond efficacy. Front. Plant Sci. 10. doi: 10.3389/fpls.2019.00845 PMC6658832 31379891

[B28] Lago J. H. G. Ramos C. S. Casanova D. C. C. Morandim A. D. A. Bergamo D. C. B. Cavalheiro A. J.. (2004). Benzoic acid derivatives from piper species and their fungitoxic activity against cladosporium cladosporioides and c. sphaerospermum. J. Nat. Prod. doi: 10.1021/np030530j 15568762

[B29] Lastochkina O. Seifikalhor M. Aliniaeifard S. Baymiev A. (2019). Bacillus spp.: Efficient biotic strategy to control postharvest diseases of fruits and vegetables. Plants 8, 1–24. doi: 10.3390/plants8040097 PMC6524353 31013814

[B30] Manimaran M. Gopal J. V. Kannabiran K. (2017). Antibacterial activity of *Streptomyces* sp. VITMK1 isolated from mangrove soil of pichavaram, Tamil nadu, India. Proc. Natl. Acad. Sci. India Sect. B Biol. Sci. 87, 499–506. doi: 10.1007/s40011-015-0619-5

[B31] Meyer H. Liebeke M. Lalk M. (2010). A protocol for the investigation of the intracellular staphylococcus aureus metabolome. Anal. Biochem. 401, 250–259. doi: 10.1016/j.ab.2010.03.003 20211591

[B32] Mnif I. Ghribi D. (2015). Lipopeptides biosurfactants: Mean classes and new insights for industrial, biomedical, and environmental applications. Biopolymers 104, 129–147. doi: 10.1002/bip.22630 25808118

[B33] Mohamad O. A. A. Li L. Ma J.-B. Hatab S. Xu L. Guo J.-W.. (2018). Evaluation of the antimicrobial activity of endophytic bacterial populations from Chinese traditional medicinal plant licorice and characterization of the bioactive secondary metabolites produced by *Bacillus atrophaeus* against *Verticillium dahliae* . Front. Microbiol. 9. doi: 10.3389/fmicb.2018.00924 PMC5954123 29867835

[B34] Moreira R. R. Nesi C. N. May De Mio L. L. (2014). *Bacillus* spp. and *Pseudomonas* putida as inhibitors of the *Colletotrichum acutatum* group and potential to control *Glomerella* leaf spot. Biol. Control 72, 30–37. doi: 10.1016/j.biocontrol.2014.02.001

[B35] More T. T. Yadav J. S. S. Yan S. Tyagi R. D. Surampalli R. Y. (2014). Extracellular polymeric substances of bacteria and their potential environmental applications. J. Environ. Manage 144, 1–25. doi: 10.1016/j.jenvman.2014.05.010 24907407

[B36] Morita T. Tanaka I. Ryuda N. Ikari M. Ueno D. Someya T. (2019). Antifungal spectrum characterization and identification of strong volatile organic compounds produced by *Bacillus pumilus* TM-r. Heliyon 5, e01817. doi: 10.1016/j.heliyon.2019.e01817 31206088 PMC6558263

[B37] Nardello-Rataj V. Leclercq L. (2014). Encapsulation of biocides by cyclodextrins: toward synergistic effects against pathogens. Beilstein J. Org. Chem. 10, 2603–2622. doi: 10.3762/bjoc.10.273 25550722 PMC4273244

[B38] Nardello-Rataj V. Leclercq L. (2016). Aqueous solutions of didecyldimethylammonium chloride and octaethylene glycol monododecyl ether: Toward synergistic formulations against enveloped viruses. Int. J. Pharm. 511, 550–559. doi: 10.1016/j.ijpharm.2016.07.045 27452423

[B39] Ni M. Wu Q. Wang J. Liu W. C. Ren J. H. Zhang D. P.. (2018). Identification and comprehensive evaluation of a novel biocontrol agent *Bacillus atrophaeus* JZB120050. J. Environ. Sci. Heal. Part B 53, 777–785. doi: 10.1080/03601234.2018.1505072 30199317

[B40] Numan M. Shah M. Asaf S. Ur Rehman N. Al-Harrasi A.. (2022). Bioactive compounds from endophytic bacteria bacillus subtilis strain EP1 with their antibacterial activities. Metabolites 12 (12), 1228. doi: 10.3390/metabo12121228 36557265 PMC9788538

[B41] Ojinnaka C. M. Nwachukwu K. I. Ezediokpu M. N. (2016). The chemical constituents and bioactivity of the seed (Fruit) extracts of *Buchholzia coriacea engler* (Capparaceae). J. Appl. Sci. Environ. Manage. 19, 795. doi: 10.4314/jasem.v19i4.29

[B42] Olanrewaju O. S. Glick B. R. Babalola O. O. (2017). Mechanisms of action of plant growth promoting bacteria. World J. Microbiol. Biotechnol. 33, 197. doi: 10.1007/s11274-017-2364-9 28986676 PMC5686270

[B43] Oliva J. Messal M. Wendt L. Elfstrand M. (2017). Quantitative interactions between the biocontrol fungus *Phlebiopsis gigantea*, the forest pathogen *Heterobasidion annosum* and the fungal community inhabiting Norway spruce stumps. For. Ecol. Manage. 402, 253–264. doi: 10.1016/j.foreco.2017.07.046

[B44] Ongena M. Duby F. Jourdan E. Beaudry T. Jadin V. Dommes J.. (2005). *Bacillus subtilis* M4 decreases plant susceptibility towards fungal pathogens by increasing host resistance associated with differential gene expression. Appl. Microbiol. Biotechnol. 67, 692–698. doi: 10.1007/s00253-004-1741-0 15578181

[B45] Rashid U. Yasmin H. Hassan M. N. Naz R. Nosheen A. Sajjad M.. (2022). Drought-tolerant *Bacillus megaterium* isolated from semi-arid conditions induces systemic tolerance of wheat under drought conditions. Plant Cell Rep. 41 (3), 549–569. doi: 10.1007/s00299-020-02640-x 33410927

[B46] Shafi J. Tian H. Ji M. (2017). Bacillus species as versatile weapons for plant pathogens: a review. Biotechnol. Equip. 31, 446–459. doi: 10.1080/13102818.2017.1286950

[B47] Shoaib A. Awan Z. A. Khan K. A. (2019). Intervention of antagonistic bacteria as a potential inducer of disease resistance in tomato to mitigate early blight. Sci. Hortic. 252, 20–28. doi: 10.1016/j.scienta.2019.02.073

[B48] Singh T. Wahla V. (2018). GC-MS analysis of antifungal compounds derived from soil actinobacteria. Int. Res. J. Phar. 9, 81–84. doi: 10.7897/2230-8407.09232

[B49] Syed-Ab-Rahman S. F. Carvalhais L. C. Chua E. Xiao Y. Wass T. J. Schenk P. M. (2018). Identification of soil bacterial isolates suppressing different *Phytophthora* spp. and promoting plant growth. Front. Plant Sci. 9. doi: 10.3389/fpls.2018.01502 PMC6201231 30405657

[B50] Syed-Ab-Rahman S. F. Xiao Y. Carvalhais L. C. Ferguson B. J. Schenk P. M. (2019). Suppression of *Phytophthora capsici* infection and promotion of tomato growth by soil bacteria. Rhizosphere 9, 72–75. doi: 10.1016/j.rhisph.2018.11.007

[B51] Tangjitjaroenkun J. (2018). Evaluation of antioxidant, antibacterial, and gas chromatography-mass spectrometry analysis of ethyl acetate extract of *Streptomyces omiyaensis* SCH2. Asian. J. Pharm. Clin. Res. 11, 271. doi: 10.22159/ajpcr.2018.v11i7.25692

[B52] Theng B. Korpenwar A. N. (2015). Phytochemical analysis of ethanol extract of *Ampelocissus latifolia* (Roxb.) *Planch tuberous* root using UV-VIS, FTIR and GC-MS. Int. J. Pharm. Sci. Res. 6 (9), 3936–3942. doi: 10.13040/IJPSR.0975-8232.6(9).3936-42

[B53] Umaiyambigai D. Saravanakumar K. Adaikala Raj G. (2017). Phytochemical profile and antifungal activity of leaves methanol extract from the *Psydrax dicoccos* (Gaertn) teys. & binn. rubiaceae family. Int. J. Pharmacol. Phytochem. Ethnomed. 7, 53–61. doi: 10.18052/www.scipress.com/IJPPE.7.53

[B54] Wang X. Q. Zhao D. L. Shen L. L. Jing C. L. Zhang C. S. (2018). “Application and mechanisms of bacillus subtilis,” in Biological control of plant disease. Ed. Meena V. S. (Singapore: Springer Singapore), 225–250. doi: 10.1007/978-981-10-8402-7

[B55] Yánez-Mendizábal V. Usall J. Viñas I. Casals C. Marín S. Solsona C.. (2011). Potential of a new strain of *Bacillus subtilis* CPA-8 to control the major postharvest diseases of fruit. Biocontrol Sci. Technol. 21, 409–426. doi: 10.1080/09583157.2010.541554

[B56] Yu J.-H. Choi G.-J. Lim H.-K. Kim H.-T. (2009). Surfactants effective to the control of cucumber powdery mildew. J. Appl. Biol. Chem. 52, 195–199. doi: 10.3839/jabc.2009.033

[B57] Zhang B. Li X. L. Fu J. Li N. Wang Z. Tang Y. J.. (2016). Production of acetoin through simultaneous utilization of glucose, xylose, and arabinose by engineered *Bacillus subtilis* . PloS One 11, e0159298. doi: 10.1371/journal.pone.0159298 27467131 PMC4965033

