# Supplementary material for: Antifungal potential of volatiles produced by Bacillus subtilis BS-01 against Alternaria solani in Solanum lycopersicum
Source: Front Plant Sci. 2023 Jan 26;13:1089562. doi: 10.3389/fpls.2022.1089562 (PMC9909239; doi:10.3389/fpls.2022.1089562)
Supplement: Supplementary file 1 [file DataSheet_1.docx]

**Supplementary Material**

**Fig. S1.** Chromatogram showing peak height of the *n*-hexane fraction of extracellular metabolites of *Bacillus subtilis* (BS-01) through GC-MS analysis.

**Fig. S2.** Chromatogram of the dichloromethane fraction of extracellular metabolites of *Bacillus subtilis* (BS-01) through GC-MS analysis.

**Fig. S3.** Chromatogram of the ethyl acetate fraction of extracellular metabolites of *Bacillus subtilis* (BS-01) through GC-MS analysis.

**Fig. S4.** Chromatogram of *n*-hexane fraction of intracellular metabolites of *Bacillus subtilis* (BS-01) through GC-MS analysis.

**Fig. S5.** Chromatogram of the dichloromethane fraction of intracellular metabolites of *Bacillus subtilis* (BS-01) through GC-MS analysis.

**Fig. S6.** Chromatogram of the ethyl acetate fraction of intracellular metabolites of *Bacillus subtilis* (BS-01) through GC-MS analysis.
